# Expedited transport versus continued on-scene resuscitation for refractory out-of-hospital cardiac arrest: A systematic review and meta-analysis

**DOI:** 10.1016/j.resplu.2023.100482

**Published:** 2023-10-07

**Authors:** Brian Burns, Henry R. Hsu, Anthony Keech, Yating Huang, David H. Tian, Andrew Coggins, Mark Dennis

**Affiliations:** a Faculty of Medicine and Health, University of Sydney, Australia; b New South Wales Ambulance, Sydney, Australia; c Westmead Hospital, Westmead, NSW, Australia; d Department of Cardiology, Royal Prince Alfred Hospital, Sydney, Australia; e Department of Anaesthesia and Perioperative Medicine, Westmead Hospital, Sydney, Australia; f Department of Surgery, Royal Melbourne Hospital, Melbourne, Australia; g The George Institute for Global Health, Sydney, Australia

**Keywords:** Heart arrest, Cardiac Arrest, Out-of-hospital, Out of hospital, Expedited transport, Transport of patients, Patient transport, Emergency Medical Services, Humans, Adults, Cardiopulmonary Resuscitation, Treatment outcome, Neurological outcome, Survival, Curb to care, Scoop and run, Load and go, Stay and play

## Abstract

**Background:**

The benefit of rapid transport from the scene to definitive in-hospital care versus extended on-scene resuscitation in out-of-Hospital Cardiac Arrest (OHCA) is uncertain.

**Aim:**

To assess the use of expedited transport from the scene of OHCA compared with more extended on-scene resuscitation of out-of-hospital cardiac arrest in adults.

**Methods:**

A systematic search of the literature was conducted using MEDLINE, Embase, and SCOPUS. Randomised control trials (RCTs) and observational studies were included. Studies reporting transport timing for OHCA patients with outcome data on survival were identified and reviewed. Two investigators assessed studies identified by screening for relevance and assessed bias using the ROBINS-I tool. Studies with non-dichotomous timing data or an absence of comparator group(s) were excluded. Outcomes of interest included survival and favourable neurological outcome. Survival to discharge and favourable neurological outcome were meta-analysed using a random-effects model.

**Results:**

Nine studies (eight cohort studies, one RCT) met eligibility criteria and were considered suitable for meta-analysis**.** On pooled analysis, expedited (or earlier) transfer was not predictive of survival to discharge (odds ratio [OR] 1.16, 95% confidence interval [CI] 0.53 to 2.53, I^2^ = 99%, *p* = 0. 65) or favorable neurological outcome (OR 1.06, 95% CI 0.48 to 2.37, I^2^ = 99%, *p* = 0.85). The certainty of evidence across studies was assessed as very low with a moderate risk of bias. Region of publication was noted to be a major contributor to the significant heterogeneity observed amongst included studies.

**Conclusions:**

There is inconclusive evidence to support or refute the use of expedited transport of refractory OHCA.

## Introduction

Annually, an estimated 350,000 patients suffer an Out-of-hospital Cardiac Arrest (OHCA) in the United States.^1^, ^2^ Despite resuscitation efforts by emergency medical services (EMS), refractory cardiac arrest (r-OHCA) frequently occurs in this group. R-OHCA is defined as failure to promptly obtain return of spontaneous circulation (ROSC) and is associated with a poor prognosis. Further, there is uncertainty regarding the timing of transport to the hospital for these patients.^3^ High-performance cardiopulmonary resuscitation (hpCPR) can result in better outcomes but a fraction of patients remain refractory to pre-hospital interventions.^4^ Where ROSC is not achieved, intra-arrest transport to a hospital may allow provision of advanced interventions such as extracorporeal membrane oxygenation cardiopulmonary resuscitation (ECPR).^5^

Recent trials studying assessments of bundles of care that include early transfer from scene to hospital for ECPR and coronary angiography have been conflicting.^6^, ^7^, ^8^ Notably, ECPR is only available in a small number of hospitals and jurisdictions. The limited reach of ECPR within the broad OHCA population emphasizes the urgency of understanding best practices in terms of decisions to transport to a hospital versus continuing hpCPR at the scene. Moreover, transport of patients with OHCA can degrade CPR quality and despite the availability of mechanical cardiopulmonary resuscitation (m-CPR), represents a significant logistical challenge.^9^, ^10^, ^11^, ^12^

Guidelines^13^, ^14^, ^15^ neither provide clear guidance on the duration of CPR that should be provided on-scene, nor the amount of time EMS should stay on-scene before transport. Therefore, the objective of this systematic review and meta-analysis was to assess the benefit of expedited transport of patients in r-OHCA against more extended on-scene resuscitation.

## Methods

### Protocol and registration

This systematic review followed the Preferred Reporting Items for Systematic Reviews and Meta-Analyses (PRISMA) guidelines.^16^ The PRISMA checklist is provided in the Supplementary Contents. The protocol and amendments were prospectively submitted to the International Prospective Register of Systematic Reviews (PROSPERO) (CRD42022310859).^17^ The protocol is provided in the Supplementary appendix. Covidence (https://www.covidence.org) was used to manage the screening, full-text review, and data extraction of studies included in the present meta-analysis.^18^

### Key definitions

*Process definition:* we categorized patient exposure groups in which authors explicitly described patient groups as rapid or expedited transport versus delayed/extended/prolonged on-scene treatment as utilizing a ‘process definition’, for purposes of analysis. For all other papers where this wasn’t explicitly stated, we applied the time cut-offs described below.

*Time definition:* As the concept of ‘expedited transport’ is not defined in the literature, we defined ‘expedited transport’ as the transport of an OHCA patient from the scene of the arrest, with the explicit intent of rapidly transporting the patient to hospital. We prospectively defined on-scene time cut-offs of <20 min as expedited and ≥20 min as standard, based on EMS systems' current operating protocols of minimum duration of resuscitation.^14^, ^19^, ^20^ Furthermore, at 20 minutes of continued resuscitation, the likelihood of survival is reported to be very low.^14^, ^20^, ^21^ More extended on-scene resuscitation was defined as an on-scene ≥20 min or not explicitly defined as expedited transport. Where published on-scene time cut-offs were not able to be directly dichotomized with this cutoff, best efforts were made to allocate studied groups as per this definition or excluded as per joint decision between investigators.

### Study eligibility

Randomised trials, non-randomised controlled trials, and observational studies (cohort studies and case-control studies) were included. We included studies that were not primarily in English if they were accompanied by an English translation.

We excluded studies including initial cardiac rhythm of asystole; traumatic cardiac arrest; paediatric populations; mixed paediatric and adult populations; studies not reporting outcomes of interest; non-human studies; ecological studies; case series; case reports review articles; conference abstracts; editorials; comments; letters to the editor. Non-English articles without an accompanying English translation were excluded.

### Search strategy

A systematic literature search was conducted using MEDLINE, Embase, and SCOPUS from dates of database inception up to 30 November 2022. The search strategy was developed in consultation with a medical librarian, for the following terms including both Medical Subject Headings (MESH) and keywords and their derivatives: (out of hospital cardiac arrest OR out-of-hospital cardiac arrest OR OHCA OR OOHCA) AND (transportation of patients OR transport* of patient* OR patient transport*) AND (cardiopulmonary resuscitation OR CPR) AND (survival rate OR survival analysis OR patient discharge). De-duplication of articles was done using EndNote X9 software^22^ and Covidence.

### Study selection

We utilised a two-step eligibility and selection process. First, two authors (HH and AC) independently screened the titles and abstracts of the de-duplicated list of articles according to predefined criteria in a blinded fashion. Articles were screened using the following inclusion criteria: studies of adult patients experiencing OHCA and that included data on the expedited transport of OHCA patients to hospital, and that compared expedited transport with usual care and reported outcomes of interest. Articles were excluded or included where both agreed. Disputes were resolved by a third author (BB). One additional author (YH) reviewed all excluded articles and found no further articles for inclusion.

### Outcome measures

In contrast to the prior PROSPERO registration primary outcomes of interest were survival to 30 days and/or hospital discharge, and favourable neurological outcome at 30 days and/or hospital discharge. Favourable neurological outcome was defined as Cerebral Performance Category (CPC) 1 or 2, or modified Rankin scale (mRS) 0, 1, or 2. The change in outcomes from PROSPERO Registration was required after the initial screening phase identified significant heterogeneity amongst included studies, leading to a limitation of meaningful outcomes that could be presented in aggregate. The presented outcomes are consistent with a large body of literature in out-of-hospital cardiac arrest and resuscitation, (1–3) and are core outcomes expected by clinicians. Outcomes of interest were reported using odds ratio.

### Risk of bias and certainty of evidence assessment

Two investigators (BB and HH) independently assessed the risk of bias of included studies. The risk of bias of randomised controlled trials was evaluated using the Cochrane risk-of-bias tool for randomised trials (RoB 2). Risk of bias of non-randomised trials was assessed using the ROBINS-I tool.^23^ Graphical presentations of the risk of bias assessments were prepared using Robvis,^24^ an open-access web-based visualization tool (https://www.riskofbias.info/welcome/robvis-visualization-tool). Disagreement in the risk of bias assessment was resolved by discussion between two investigators. Bias was assessed per study rather than per outcome. Certainty of overall evidence across studies was assessed using the GRADE methodology.^25^

### Data extraction

We used a pre-defined standardised data extraction form on Covidence to extract data from the included studies. Observational outcome headings extracted included: authors; year of publication; study design; study population; demographics and geographical origin. Where propensity-matched data was available in observational studies, these were preferred over whole cohort data.

### Statistical analysis

Meta-analysis was performed with the Hartung-Knapp random-effects model to account for variations in regional protocols for studies that directly compared expedited and standard transport. Random-effects meta-analysis of proportions were used to combine single-arm binary outcomes. Treatment effects are presented with odds ratio (95% confidence interval). I^2^ statistic was used to estimate the proportion of total variability attributable to heterogeneity rather than to sampling error. Thresholds for I^2^ values for low, moderate, and high heterogeneity were considered as 0–49%, 50–74% and ≥75%, respectively. The region of study was assessed with meta-regression as a potential source of heterogeneity between studies, as it was hypothesised *a priori* that clinical practices in Western countries may be different to those from Asian/Eastern countries. Further meta-regression was performed on the number of recruited patients and the median year of recruitment. Publication bias was assessed by Egger’s test if at least ten studies were identified.^26^, ^27^ Outliers were assessed with leave-one-out analysis and Baujat plots. All p-values were 2-sided; p values <0.05 were considered statistically significant. All statistical analyses were conducted in R version 4.0.2 (R Foundation for Statistical Computing, Vienna, Austria), with packages *meta* (version 6.1–0) and *metafor* (version 3.8–1). Bayesian meta-analysis was also performed as a sensitivity analysis, with µ as a normal distribution N(0,10000), and τ as half-normal distribution HC(0, 0.5). A second analysis that incorporated predictive heterogeneity derived from the Cochrane Database of Systematic Reviews for τ was also performed.^28^ Results are presented as credible intervals (CrI).

## Results

### Study selection

**Fig. 1:**
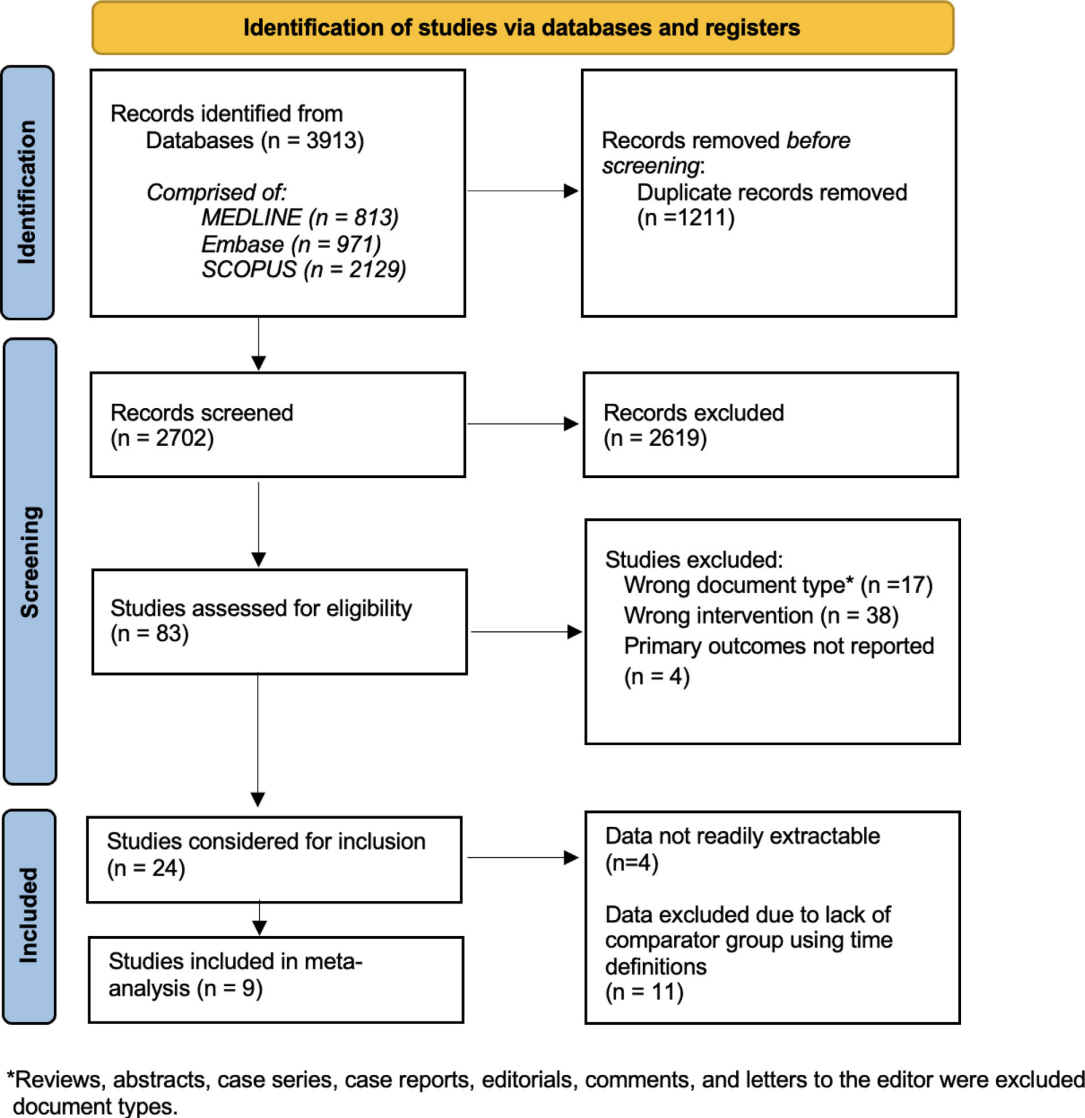
Preferred Reporting Items for Systematic Reviews and Meta-Analyses (PRISMA) flowchart of systematic search and study selection.

The search strategy identified 3,913 records of which 83 records were eligible for full text review. The PRISMA diagram of the study selection process is shown in Fig. 1. Nine studies reported data that met the definition of expedited transport; seven were classified according to the time definition while the remaining two were classified using the process definition. Four studies were conducted in Asia, three in Europe, and two in North America. An overview of included studies is provided in Table 1, Table 2. Studies excluded due to a lack of a appropriate comparator group are summarized in Supplementary Table 2.

**Table 1:** Characteristics of interventions and outcomes of studies included in meta-analysis.

**Study**	**Year**	**N**	**Country**	**Study Design**	**Age**	**Male (%)**	**Risk of Bias**
**Time definition**							
Holmberg^34^	2022	174	Denmark	Retrospective Cohort	56	69%	Low
Berry^29^	2021	145,153	United States	Retrospective Cohort	61	62%	Moderate
Jang^30^	2021	3,340	Korea	Retrospective Cohort	63	67%	Moderate
Park^31^	2021	344	Korea	Retrospective Cohort	59	82%	Moderate
de Graaf^35^	2019	655	Netherlands	Retrospective Cohort	63	79%	Moderate
Kim^33^	2017a	16,974	Korea, Japan, Singapore, Taiwan	Retrospective Cohort	71	63%	Moderate
Kim^32^	2017b	41,054	Korea	Retrospective Cohort	NR	64%	Moderate
**Process definition**							
Belohlavek^7^	2022	256	Czech Republic	RCT	58	83%	Moderate
Grunau^6^	2020	43,132	United States and Canada	Retrospective Cohort	66	65%	Low
Lim^47^	2020	25,895	Singapore and Australia	Retrospective Cohort	66	67%	Moderate

**Table 2:** Subgroup characteristics. Survival/CPC data presented as N (%).

**Study**	**Group classification**	**Subgroup**	**N**	**Survival to D/C**	**Survival to 30 days**	**CPC 1**–**2 at discharge**	**CPC 1**–**2 at 30 days**	
**Time definition**								
Holmberg 2022	Expedite	Intra-cardiac arrest transportwithin 20 minutes	87	NR	31 (35.6)	NR	NR	
Holmberg 2022	Standard	On-scene resuscitation	87	NR	20 (23.0)	NR	NR	
Berry 2021	Expedite	STI < 19.3 mins	47,511	5449 (11.5)	NR	4248 (8.9)	NR	
Berry 2021	Standard	STI 21.9–25.1 mins	41,532	7554 (18.2)	NR	6174 (14.9)	NR	
Berry 2021	Standard	STI > 25.1 mins	29,548	6613 (22.4)	NR	5580 (18.9)	NR	
Jang 2021	Expedite	STI < 19 min	1,670	266 (15.9)	NR	175 (10.5)	NR	
Jang 2021	Standard	STI > 19 mins	1,670	133 (8)	NR	71 (4.3)	NR	
Park 2021	Expedite	STI < 15 mins	151	60 (39.7)	NR	NR	NR	
Park 2021	Standard	STI > 15 mins	193	38 (19.7)	NR	NR	NR	
de Graaf 2019	Expedite	STI < 20 mins	178	NR	13 (7.3)	NR	NR	
de Graaf 2019	Standard	STI 20–30 mins	282	NR	12 (4.3)	NR	NR	
de Graaf 2019	Standard	STI > 30 mins	195	NR	4 (2.1)	NR	NR	
Kim 2017a	Expedite	STI < 8 mins	4,432	459 (10.4)	NR	208 (4.7)	NR	
Kim 2017a	Expedite	STI 9–15 mins	7,551	485 (6.4)	NR	238 (3.2)	NR	
Kim 2017a	Standard	STI > 16 mins	4,991	245 (4.9)	NR	128 (2.6)	NR	
Kim 2017b	Expedite	STI < 4 mins	7,348	458 (6.2)	NR	217 (3)	NR	
Kim 2017b	Expedite	STI 4–8 mins	15,110	882 (5.8)	NR	484 (3.2)	NR	
Kim 2017b	Expedite	STI 8–12 mins	11,415	596 (5.2)	NR	348 (3)	NR	
Kim 2017b	Standard	STI 12–60 mins	7,181	351 (4.9)	NR	207 (2.9)	NR	
**Process definition**								
Belohlavek 2022	Expedite	Intra-arrest transport with ongoing m-CPR	124	NR	54 (43.5%)	NR	38 (30.6%)	
Belohlavek 2022	Standard	Continued ACLS on-scene	132	NR	45 (34.1%)	NR	24 (18.2%)	
Grunau 2020	Standard	Continued on-scene resuscitation	18,299	1557 (8.5)	NR	NR	NR	
Grunau 2020	Expedite	Intra-arrest transport	9,406	372 (4)	NR	NR	NR	
Grunau 2020	Standard	Continued on-scene resuscitation	10,324	NR	NR	733 (7.1)	NR	
Grunau 2020	Expedite	Intra-arrest transport	5,103	NR	NR	148 (2.9)	NR	

NR, not reported; STI, scene-time interval.

**Fig. 2:**
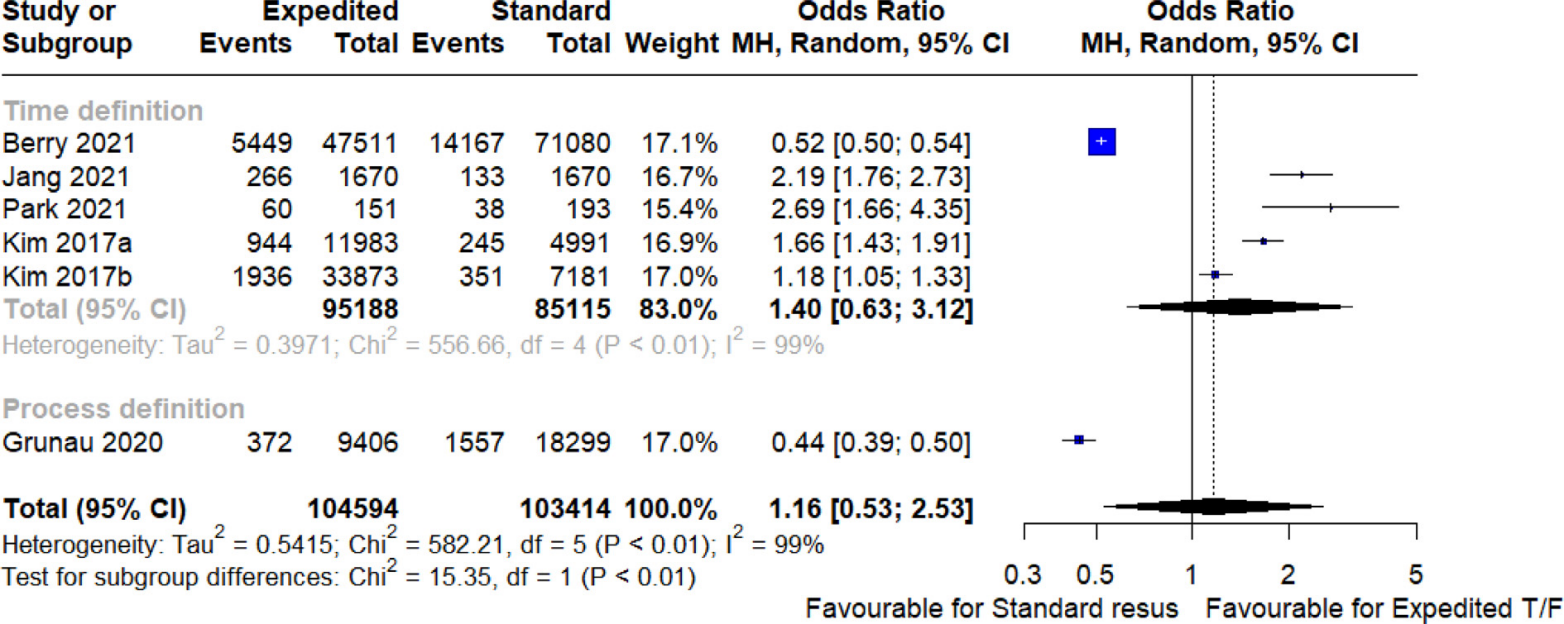
Survival to discharge. *Cardiac recovery at 30 days.

**Fig. 3:**
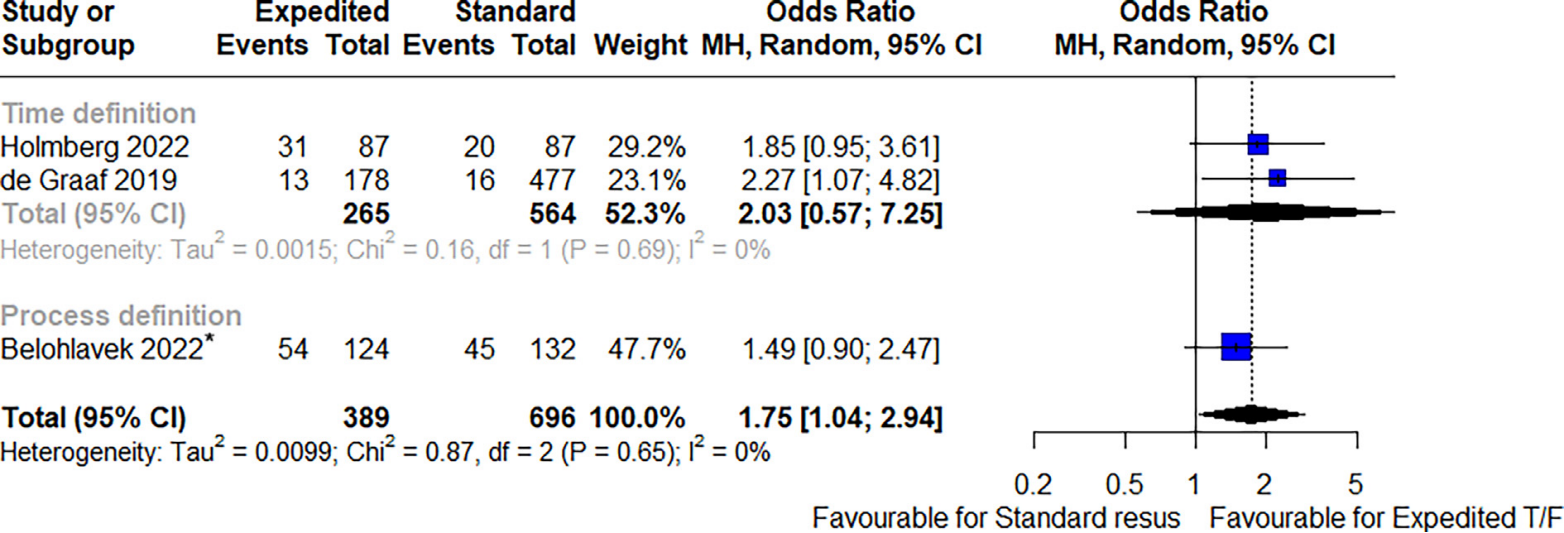
Survival to 30 days. *CPC 1–2 reported at 30 days.

**Fig. 4:**
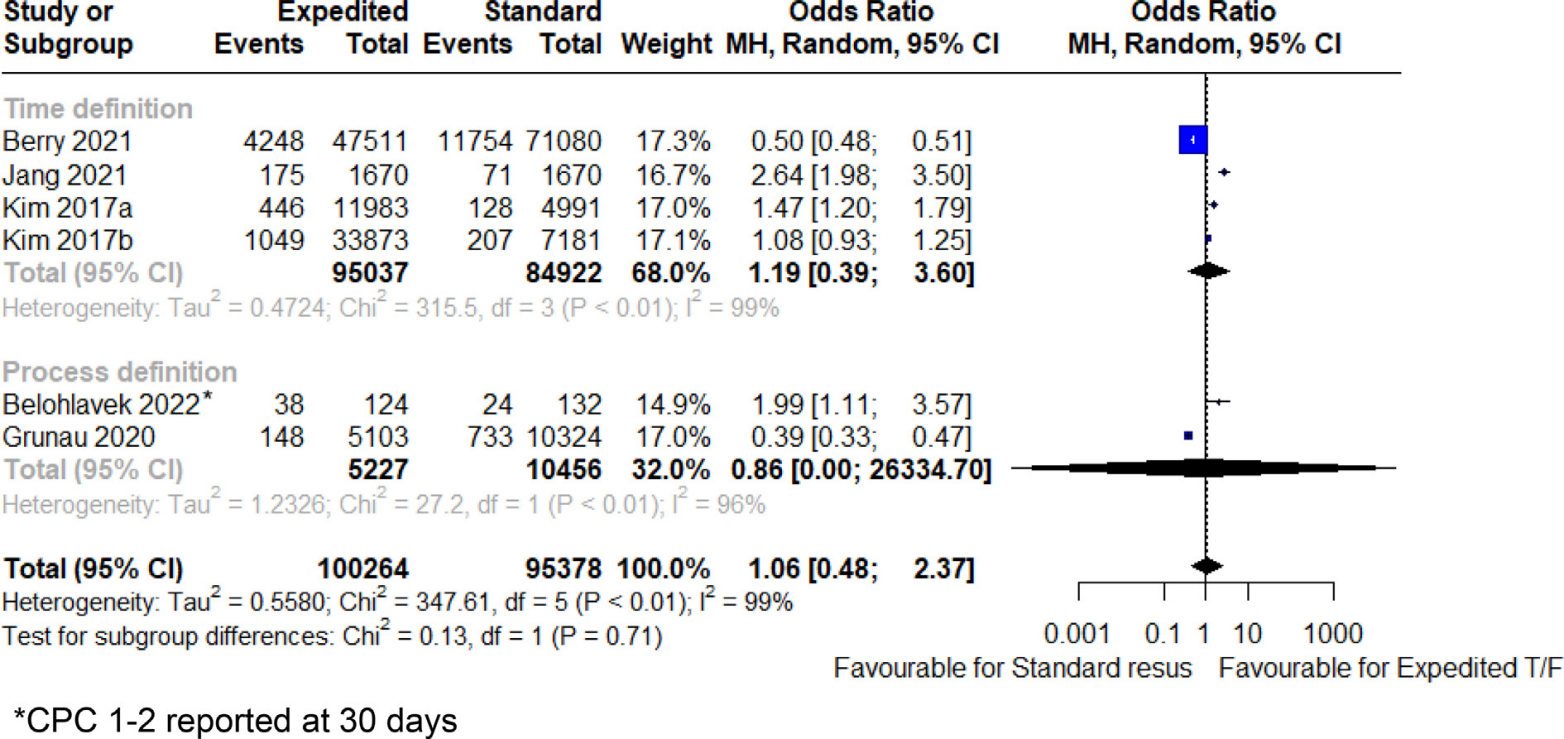
CPC 1–2 on discharge.

**Fig. 5:**
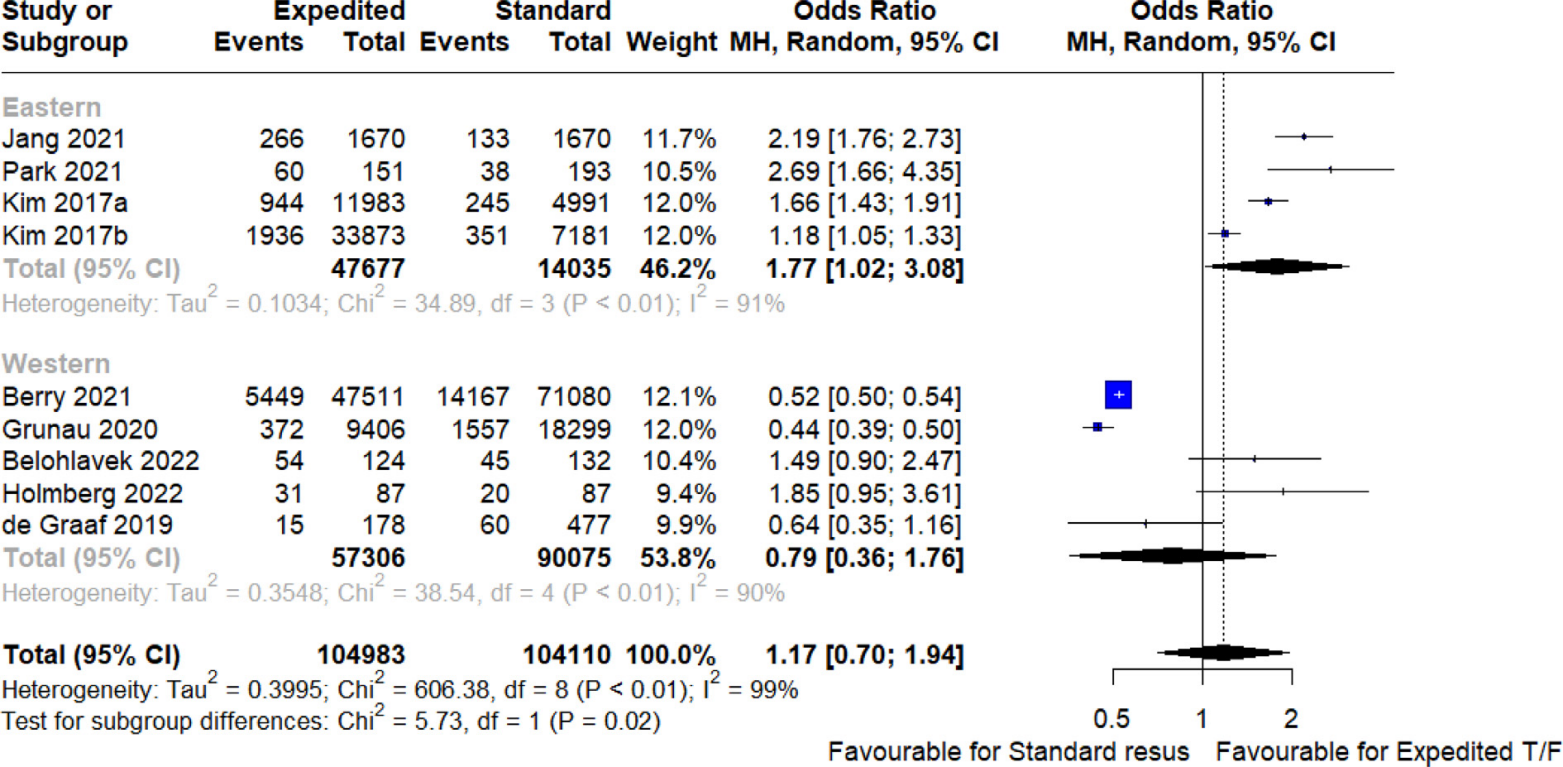
Effect of region of publication on survival to discharge or 30 days.

The mean age amongst studies that reported age was 62.9 years. Six studies reported survival to hospital discharge,^6^, ^29^, ^30^, ^31^, ^32^, ^33^ three studies reported survival to 30 days.^7^, ^34^, ^35^ Favourable neurological status on discharge was reported by six studies.^6^, ^7^, ^29^, ^30^, ^32^, ^33^

### Clinical outcomes

The effect of the continued onscene resuscitatio or expedited on clinical outcomes, of included studies are shown in (Fig. 1, Fig. 2, Fig. 3, Fig. 4, Fig. 5). Overall survival to discharge, survival to 30 days, and having favourable neurological survival was 10.9% (104594 patients), 24.5% (389 patients), and 6.9% (100264 patients), respectively, for cohorts that were classified as receiving expedited transport. The lower percentage of survival to discharge days than survival to 30 days is an unexpected finding, likely a reflection of the fewer studies (and thus smaller population size) that reported the outcome of survival to 30 days. For cohorts that met the definition of on-scene resuscitation, it was 9.4% (103414 patients), 14.8% (696 patients), and 6.5% (95378 patients), respectively.

There was no significant benefit in survival to discharge overall for those who received expedited versus standard care [odds ratio 1.16 (95% CI 0.53 to 2.53), I^2^ = 99%, *p* = 0.65, 6 studies, 208,008 patients]. Bayesian meta-analysis identified similarly credible intervals that crossed unity (OR 1.15, 95% CrI 0.76 to 1.76, Supplementary Table 4).

Survival to 30 days was favourable for the expedited transport group but should be interpreted with caution given the limited sample size [odds ratio 1.75 (95% CI 1.04 to 2.94), I^2^ = 0%, *p* = 0.04, 3 studies, 1085 patients]. No difference was seen when both survival to discharge and survival to 30 days were combined as a single metric (OR 1.17 (95% CI 0.7 to 1.94), *p* = 0.50).

Favourable neurological outcome (CPC 1–2) on discharge was not different in either group [odds ratio 1.06 (95% CI 0.48 to 2.37), I^2^ = 99%, *p* = 0.85, 6 studies, 195,642 patients]. Similar results were seen with Bayesian meta-analysis (OR 1.06, 95% CrI 0.69 to 1.63).

### Heterogeneity analysis – Region of publication

When 30-day survival was separated by region of EMS system into Eastern hemisphere (Asian studies) and Western hemisphere, a borderline statistically significant effect was seen that favoured expedited transport in Eastern hemisphere studies, not seen in Western studies, but without any overall effect seen in aggregate.

There was significant heterogeneity by region of publication: Eastern hemisphere (Asian) studies found a more pronounced effect size (earlier intra-arrest transport) than the western cohorts (β = −0.839, *p* = 0.05). This accounted for 35% of overall heterogeneity. Baujat plot of study influence is presented in Supplementary Fig. 2. No significant influence was seen of either sample size or median year of patient recruitment.

### Risk of bias

**Fig. 6:**
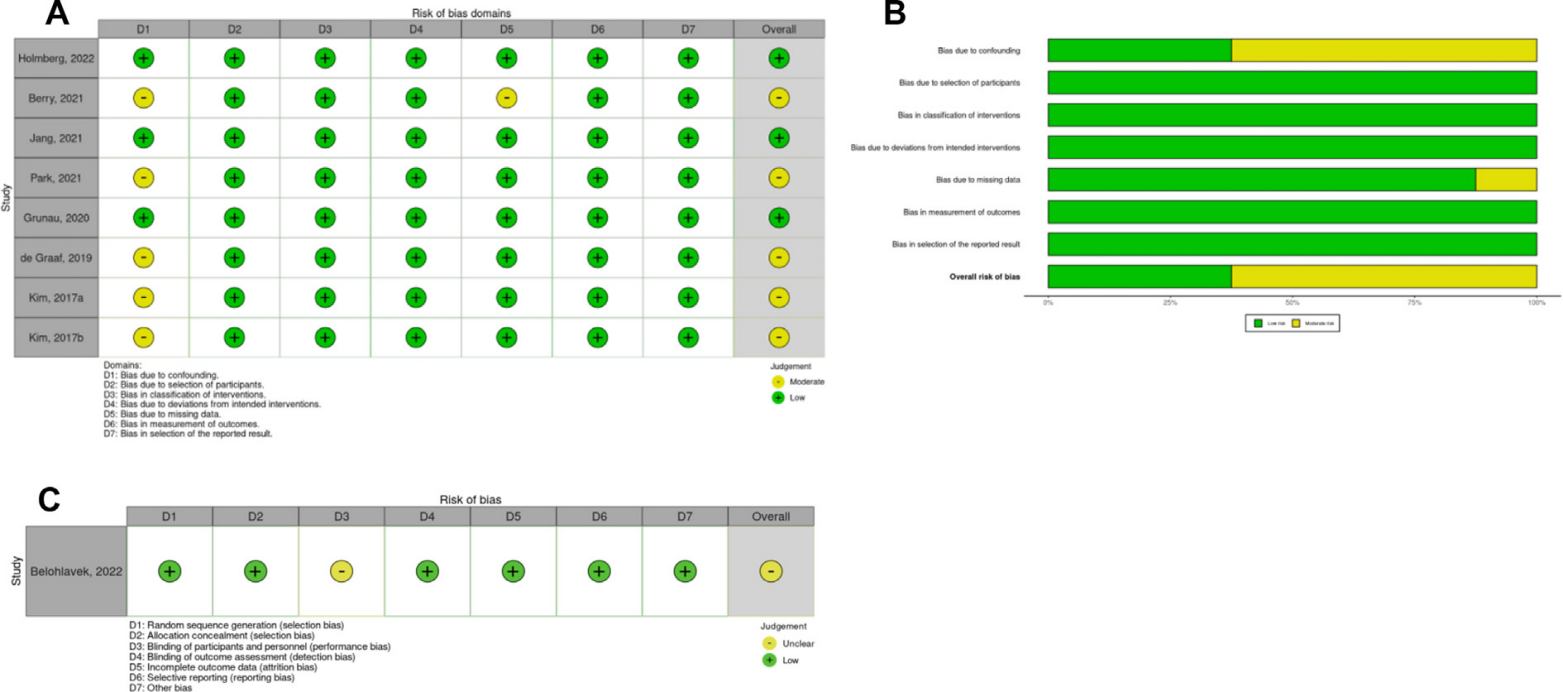
Risk of bias assessments for included studies presented using A) ROBINS-I instrument for each observational study B) summary of ROBINS-I domains across observational studies C) Cochrane RoB 2 instrument for one randomized controlled trial.

The risk of bias within individual studies was assessed as moderate, due to residual confounding variables not adjusted by authors of included studies (Fig. 6). The remaining ROBINS-1 domains were all judged to be of low risk of bias. For the sole RCT, a moderate risk of bias was noted due to lack of blinding. Egger’s test was not performed as only nine studies were identified, though inspection of funnel plot suggested imbalance favouring standard protocols (Supplementary Fig. 3). Leave one out analysis did not identify any studies which changed the direction of the overall treatment effect nor any that notably reduced overall heterogeneity.

### Certainty of evidence across studies

The overall certainty of evidence across all studies was judged to be of very. GRADE summary table is presented in Table 3.

**Table 3:** Summary of Findings Table.

**Patient or population:** adult patients experiencing out-of-hospital cardiac arrest **Setting:** community, outpatient **Intervention:** Expedited transport **Comparison:** Usual care					
**Outcomes**	**№ of participants** **(studies)**	**Certainty of the evidence** **(GRADE)**	**Relative effect** **(95% CI)**	**Anticipated absolute effects**	
				**Risk with usual care**	**Risk difference with Expedited transport**
Survival to discharge	208,008 (6 observational studies)	⊕○○○ Very low^a,b^	**OR 1.16** (0.53 to 2.53)	16 per 100	**2 more per 100** (7 fewer to 16 more)
Survival to 30 days	1,085 (2 observational studies, 1 RCT)	⊕⊕○○ Low^c^	**OR 1.75** (1.04 to 2.94)	12 per 100	**7 more per 100** (0 fewer to 16 more)
Favourable neurological outcome on discharge assessed with: CPC 1–2	195,642 (5 observational studies, 1 RCT)	⊕○○○ Very low^a,b^	**OR 1.06** (0.48 to 2.37)	135 per 1,000	**7 more per 1,000** (65 fewer to 135 more)
*The risk in the intervention group (and its 95% confidence interval) is based on the assumed risk in the comparison group and the **relative effect** of the intervention (and its 95% CI). **CI:** confidence interval; **OR:** odds ratio					
**GRADE Working Group grades of evidence** **High certainty:** we are very confident that the true effect lies close to that of the estimate of the effect. **Moderate certainty:** we are moderately confident in the effect estimate: the true effect is likely to be close to the estimate of the effect, but there is a possibility that it is substantially different. **Low certainty:** our confidence in the effect estimate is limited: the true effect may be substantially different from the estimate of the effect. **Very low certainty:** we have very little confidence in the effect estimate: the true effect is likely to be substantially different from the estimate of effect.					

*Explanations*.

a. risk of bias in several included studies from confounding.

b. Significant heterogeneity as evidenced in I^2^.

c. Small studies, small sample sizes and large error bars.

## Discussion

In this systematic review of nine studies and 224,520 patients, we did not identify significant benefit of earlier transport of patients with r-OHCA compared to more extended on-scene resuscitation. However, significant heterogeneity was present with a moderate risk of bias and overall certainty of evidence was very low.

The decision as to whether to transport a patient from the scene in r-OHCA or to continue resuscitation on-scene is challenging, with no guidelines available to inform clinical practice.^13^, ^14^, ^15^ Concerns exist that intra-arrest transport may compromise CPR quality and interfere with sustained uninterrupted hpCPR.^9^, ^10^, ^11^, ^12^, ^36^ However, prolonged on-scene resuscitation may delay definitive diagnosis and access to advanced therapies that can only be provided in hospital (e.g., coronary angiography and/or ECPR).^21^ It has also been well-documented that survival rates decline with the length of resuscitation.^37^, ^38^ These conflicting priorities highly complicate decision making in the prehospital setting.

Randomized control trials of expedited transport from scene thus far have included some bundles of care, incorporating ECPR and urgent coronary angiography^7^, ^8^, ^39^ Data from these trials are conflicting, with some reporting a clear benefit of an early transfer for ECPR strategy while others finding no benefit in utilizing an expedited bundle of care.^33^, ^7^ The recent INCEPTION trial reported no significant benefit of an ECPR strategy, though this study was complicated by long delays to ECPR initiation upon hospital arrival.^34^

Given the importance of time, an expedited transfer strategy may initially appear to be advantageous. However, even if the strategy with expedited transfer to ECPR is taken to be beneficial, there remain several questions pertaining to OHCA transfer that still need to be clarified. First, the inclusion criteria for ECPR remain restrictive, with only an estimated 4–11% of patients meeting commonly used inclusion criteria.^40^, ^41^, ^42^, ^43^ Second, ECPR is resource intensive and only offered in a small number of locations and likely to remain in limited metropolitan locations.^44^, ^45^, ^46^ For a large proportion of OHCA patients and EMS providers, especially if ECPR is not available, it remains unclear whether early transport to the hospital or staying on scene is advantageous.

Our review did not identify any randomised trial that directly compared an expedited transport strategy with a conventional strategy, and meta-analyses using pre-defined time and process definitions revealed no clear benefit in either approach. Thus, in non-ECPR-based systems, the best decision for OHCA and at which time point to execute that decision is still unclear. The currently recruiting EVIDENCE Study (ACTRN12621000668808), may provide some answers to this question. The lack of benefit of either strategy in our systematic review may be due to the heterogeneity in cardiac arrest management within and between systems, as well as the retrospectively applied definitions of expedited transfer versus extended on-scene resuscitation. Moreover, many of the studies did not report on the availability or use of mechanical CPR devices or subsequent interventions in-hospital (e.g., percutaneous coronary intervention, ECPR) that may impact outcomes.

We noted geographical location as a major source of heterogeneity. In studies from the United States and Canada, survival was favoured using extended on-scene resuscitation, while this was the opposite for studies from Korea, Japan, and Taiwan. Geography was able to account for 35% of overall heterogeneity. These differences are likely a function of culture, EMS system historical practice, operating and termination of resuscitation protocols impacting outcomes. Future studies need to more robustly report implementation criteria and definitions, as well as provide more granular data where possible to better understand the study conditions, and to assist with later comparisons and integration of results.

## Limitations

As there is no accepted definition of expedited transport, we prospectively defined on-scene time cut-offs of <20 min as expedited and ≥20 min as standard to enable analysis. Many of the studies did not comment on the availability or use of mechanical CPR devices or interventions in-hospital (percutaneous coronary intervention, ECPR). The relevant literature in this review largely contained observational trials, with many demonstrating risks of bias from confounders. Furthermore, the sole RCT was limited by the lack of blinding of treatment received during outcome ascertainment. Nonetheless, we have demonstrated that the current literature contains conflicting evidence and generally low evidence on the benefit of expedited transport for r-OHCA.

## Conclusion

Expedited transfer from scene of cardiac arrest was not associated with increased survival from cardiac arrest when compared to more prolonged time spent on scene, with significant study and geographical heterogeneity present.

## Funding

Dr Keech is supported by an NHMRC Investigator grant. Dr Dennis is supported by a Post-Doctoral Scholarship (Ref: 105849) from the National Heart Foundation of Australia. The National Heart Foundation had no role in the study design, collection, analysis or interpretation of the data nor in writing of the data and submission of the article.

## Credit Author statement

All authors in this manuscript have given substantial contribution to all of the following:

the conception and design of the study, or acquisition of data, or analysis and interpretation of data,
drafting the article or revising it critically for important intellectual content,
Have given final approval of the version to be submitted.

## Declaration of competing interest

The authors declare that they have no known competing financial interests or personal relationships that could have appeared to influence the work reported in this paper.
